# Synthesis of novel modified acrylamide copolymers for montmorillonite flocculants in water-based drilling fluid

**DOI:** 10.1186/s13065-023-01042-w

**Published:** 2023-09-24

**Authors:** Shirin Faridi, Akbar Mobinikhaledi, Hassan Moghanian, Meisam Shabanian

**Affiliations:** 1 Department of Chemistry, Faculty of Science, Arak University, Arak, Iran; 2 Materials and Energy Research Center, Dezful Branch, Islamic Azad University, Dezful, Iran; 3 Faculty of Chemistry and Petrochemical Engineering, Standard Research Institute (SRI), P.O. Box 31745-139, Karaj, Iran

**Keywords:** Polyacrylamide, Montmorillonite, Flocculation agent, Water-based drilling fluid

## Abstract

A study was conducted to treat the water-based drilling fluid through coagulation-flocculation. Innovative modified acrylamide copolymers were utilized as montmorillonite flocculants to improve drilling performance and reduce environmental contamination. A series of acrylamide copolymers was prepared by in situ free radical polymerization in aqueous medium using ammonium persulfate as a radical initiator. The chemical structure of the prepared copolymers was confirmed by FT-IR (Fourier-Transform Infrared Spectroscopy) and the polydispersity indices of the copolymers determined using gel permeation chromatography. Thermal gravimetric analysis showed that the copolymers have a very high temperature tolerance, i.e. they are stable up to 390 °C. In this paper, acrylamide copolymers were used as coagulant with cationic, anionic groups or both of them simultaneously. Consequently, in order to clarify the relationship between inhibitive properties, sedimentation volume measurement, SEM (scanning electron microscope), XRD (X-ray diffraction) and contact angle were adopted. Some factors including molecular weight and molecular chain affecting the interaction between copolymers and clay particles were analyzed. Anionic sample with the highest molecular weight can reduce the interlayer spacing of the hydrated clay to the minimum. Amphoteric sample exhibits the best performance as a coagulant in comparison with other copolymers.

## Introduction

One of the main problems of the oil well drilling industry in the world is the swelling and absorption of water by the clay particles in the ground during drilling operations with water-based fluids, which causes a very high drilling cost [1]. The significance of clays in drilling muds lies in their capacity to achieve optimal viscosity levels with relatively low solid concentrations and to sustain the desired viscosity throughout the entire drilling operation. In the oil drilling industry one of the important minerals is montmorillonite. Montmorillonite stands as a foundation for the integrated progress of drilling operations due to its pivotal role in pressure management and lubrication, [2–4]. The use of oil-based drilling fluids is limited due to the high price of these fluids [5]. Design and production shale stabilization additives for the application in water-sensitive shaly formations is on attention [6]. The first commercially successful high molecular weight soil stabilisers were introduced in the early 1980s [7]. The thermal stability of polymers is an important factor in their efficiency as soil stabilisers in very deep oil wells, so it is necessary to design a polymer with higher thermal stability than the existing industrial samples [8–10]. Coagulation is the process of overcoming the impulsive energy barrier between particles by increasing the ionic strength [11]. The interaction between the polymer chain and clay plates is through the attraction between electric charges. Thus by changing the type and number of charged functional groups in the polymer chain, the amount of their interaction with the clay plates will change [12–18]. On the other hand, freshwater is a dwindling resource, with 3.6 out of 7.7 billion people lacking reliable sources. Water scarcity is an imminent and unavoidable crisis, as global water demand is expected to increase by 55% by 2050. As a result, a concerted effort has begun globally to reuse water and facilitate sustainable water treatment processes to meet increasingly stringent regulatory norms. Coagulation and flocculation are important phenomena that find wide applications in water purification [19–21]. In this research, by placing amphoteric, cationic and anionic groups in each of the synthetic modified acrylamide copolymer chains, we investigated the effects of changing the type and intensity of electric charge on the interaction of clay plates with the polymer chains. Finally, we proposed a model and mechanism with the most interaction between clay plates and polymer, and the intensity of these interactions has a direct relationship with their properties of inhibiting the swelling of clay particles.

## Experimental

### Materials

Acrylamide (AM) and ammonium persulfate (APS) were purchased from merck company. 3-[dimethyl-[2-(2-methylprop-2-enoyloxy)ethyl]azaniumyl]propane-1-sulfonate (DMAPS), diallyldimethylammonium chloride (DADMAC) and 2-acrylamido-2-methylpropane sulfonic acid (AMPS) were provided from Aldrich company. Purified the montmorillonite supplied by southern clay products were used. All chemicals were obtained from commercial sources and used as received without purification.

### Measurements

Infrared spectra between 400 and 4000 cm^−1^ were obtained on Galaxy series FT-IR 5000 spectrophotometer at 2 cm^−1^ resolution. The molecular weight of obtained polymers was determined by gel permeation chromatography (GPC) analysis with a K-2301 (KNAUER) detector calibrated with linear polyacrylamide standard. The thermogravimetric analysis (TGA) and differential scanning calorimeter (DSC) data for polymers were taken respectively on a Mettler TGA Q5000 TA and a Mettler DSC 2500 TA with heating rate of 10 °C/min in a N_2_ atmosphere. The XRD analysis of nanocomposites were performed by XPERT-PRO X-Ray diffractometer. The XRD diffractograms were obtained at 2θ, in the range of 5–15°.The bragg equation was used to calculate the interlayer spacing (d) nλ = 2d sinθ. The surface morphology of all samples was analyzed using scanning electron microscopy (SEM) (TESCAN, Mira 3-XMU). Contact angles were determined with using digital camera equipped on contact angle tester (JIKAN, CAG-20 SE).

### Polymerization

**Scheme 1:**
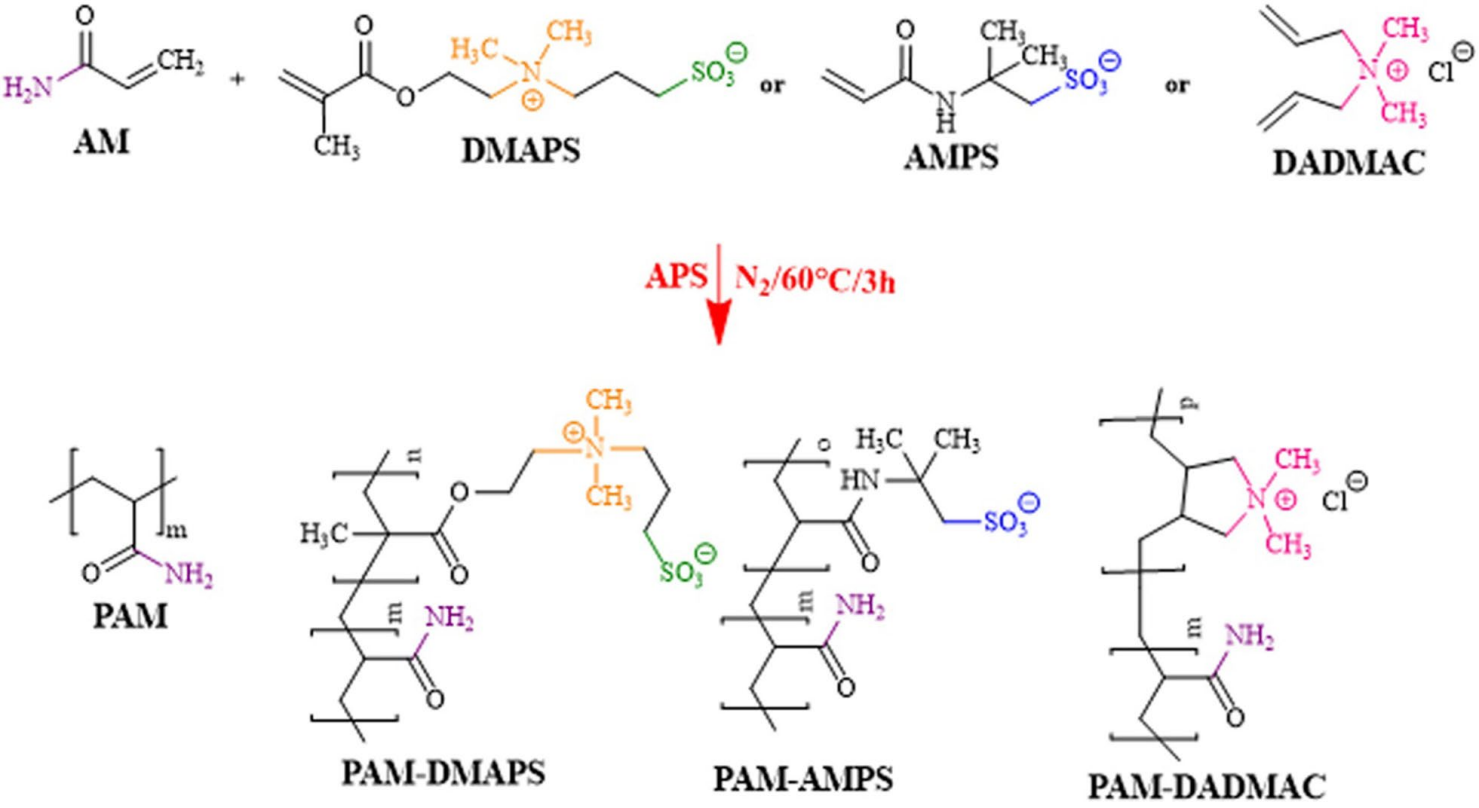
Polymerization of acrylamide and modified acrylamide copolymers

Polymer of acrylamide and copolymers-based AM were synthesized via conventional free-radical polymerization which were shown in Scheme 1. The polymerization was performed in a glass reactor, equipped with a reflux condenser, a nitrogen gas inlet and mechanical stirrer. Firstly, the glass reactor was charged with monomers and deionized water (10 wt% aqueous solution). The reactor was allowed to stand at room temperature. The solution was sprayed with N_2_ for 20 min that oxygen removed from the water. Only for the anionic sample (AMPS), it was necessary to adjust the pH of aqueous solution to around 8.5. The water soluble initiator Ammonium persulfate (APS) (0.1% mol of monomers) was dissolved in 10 mL distilled water and purged with N_2_ for 15 min was then added to the reactor. The mixture was stirred for 3 h at 60 °C. The final solution was relatively clear and highly viscous. After the completion of polymerization process, the reaction mixture was cooled and diluted with water, and then stirred gently with a mechanical stirrer until a homogeneous solution obtained. The polymerization product was mixed with acetone, so that the polymer precipitated from the original aqueous solution. Cotton-like solid dried in vacuum at 40 °C for about 6 h and was then crushed.

### Preparation of PAM and copolymers-based AM/MMT nanocomposite

A suspension of montmorillonite was poured into any graduated cylinder (0.5 g per 100 cc of water). After the addition of acrylamide copolymers with different percentages (2 and 5%) as inhibitor, MMT aggregated significantly. Then the cylinders with their contents were stirred and placed on a surface to determine the amount of sediment in a classical way. Nanocomposites were then precipitated in water, filtered and dried under vacuum overnight.

## Results and discussion

### Fourier transform infrared spectroscopy of PAM and copolymers-based AM

**Fig. 1:**
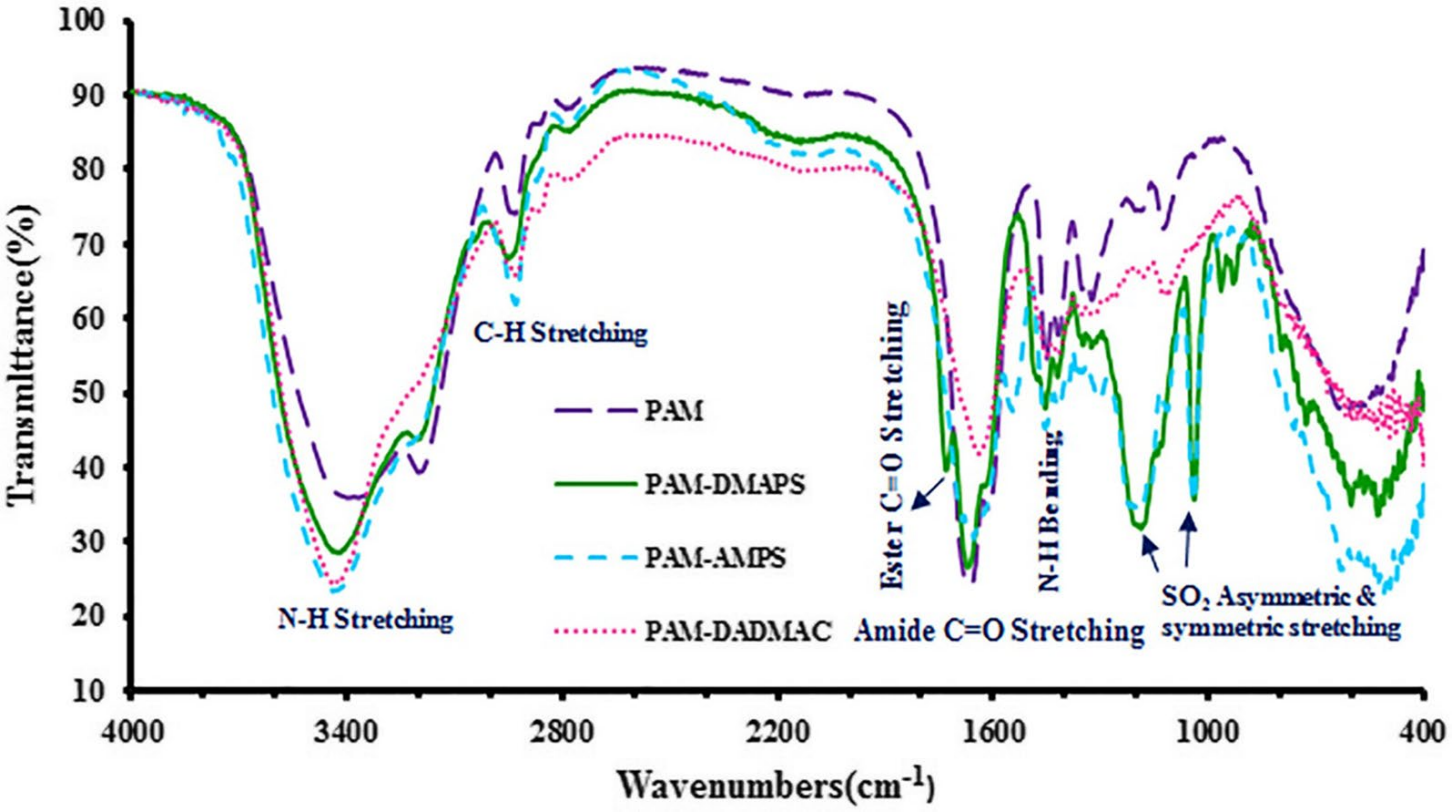
FT-IR spectra of PAM, PAM-DMAPS, PAM-AMPS and PAM-DADMAC

The FT-IR spectra of PAM and copolymers-based AM were represented in Fig. 1. The IR spectra of all products confirm the presence of amide carbonyl and N-H groups as indicated by the absorption bands at 1650 and 3400 cm^−1^, respectively [22]. It was observed that PAM-DMAPS and PAM-AMPS units exhibit several characteristics bands. The absorption bands at 1020 and 1200 cm^−1^ were assigned to the SO symmetric and asymmetric stretch of sulfonic acid groups respectively. Furthermore, the ester carbonyl band is observed at 1707 cm^−1^ exclusively in the IR spectrum of PAM-DMAPS compound [23].

### Molecular weight

As shown in Table 1, the number-average molecular weight and polydispersity index (PDI) of copolymers, which were obtained. The greater solubility of the AMPS in water caused that the molecular weight of PAM-AMPS is higher than all polymers. The number-average molecular weight of the PAM-AMPS polymer (Mn) is about 9,870,000 g/mol, Weight-average molecular weight (Mw) = 10,400,000 g/mol and Mw/Mn = 1.04. It appears that PDI is ranging between 1.04 and 3.69 indicating that the copolymers molecular weight distribution is nearly monodispers or from narrow distribution type. In the best case, the awesome polydispersity index is close to one for PAM-AMPS sample.

**Table 1:** Molecular weights and molecular weight distributions of PAM and copolymers-based AM

Polymer	Mn (g/mol)	Mw (g/mol)	PDI ^a^(Mw/Mn)
PAM	2,950,000	5,220,000	1.77
PAE-DMAPS	550,000	2,030,000	3.69
PAM-AMPS PAM-DADMAC	9,870,000 2,870,000	10,300,000 4,320,000	1.04 1.50

^a^Polydispersity index

### Thermogravimetric analysis and derivative thermogravimetry

**Fig. 2:**
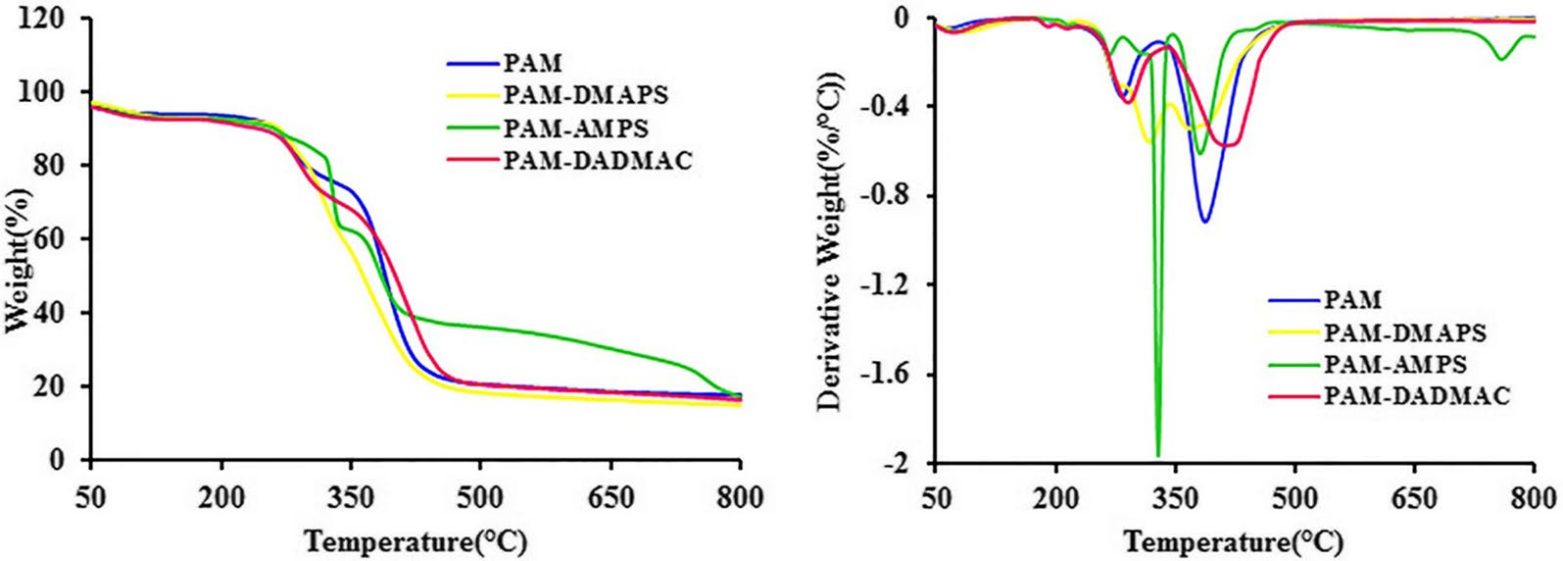
TGA and DTG curves of PAM and copolymers-based AM

The thermogravimetric analysis of PAM and copolymers-based AM are shown in Fig. 2. The thermal properties of PAM and copolymers-based AM were investigated with TGA and DTG, and thermal data are summarized in Table 2. In the case of the TGA analyses, all samples decomposition showed two distinct stages of mass loss. First stage, PAM and copolymers-based AM show a very small weight loss at 300 °C implying removal of moisture. The second stage was the polymer main chain decomposition. Probably, PAM removes water from the structure of polymers. In PAM-AMPS and PAM-DMAPS samples SO_2_ was removed. PAM-DADMAC polymer decomposition occurred at the highest temperature compared to others, because it has less N-H compared to PAM, so less water removal. The thermal decomposition temperature of PAM-AMPS is more than PAM-DMAPS, because of the strength of the C-N bond is greater than C-S. These samples exhibited good resistance to thermal decomposition and began to decompose gradually above 300 °C temperature.

**Table 2:** Thermal characteristic data of the PAM and copolymers-based AM

Polymer	T_max_(°C)^a^	Tg(°C)^b^
PAM	390	195
PAE-DMAPS	342	181
PAM-AMPS PAM-DADMAC	340 421	225 193

^a^Temperature of the maximum rate of decomposition from derivative TGA

^b^Glass transition temperature was recorded at a heating rate of 10 °C min^−1^ in a nitrogen atmosphere

### Differential scanning calorimetry

**Fig. 3:**
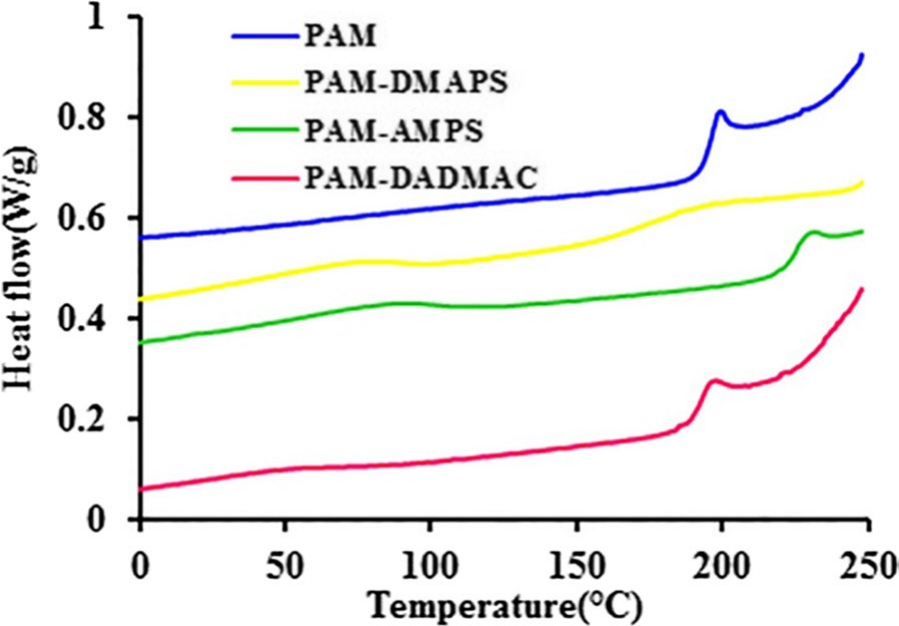
The DSC thermograms of PAM and copolymers-based AM

Figure 3 shows the DSC curves of PAM and copolymers-based AM. Initial degradation at about 100 °C corresponds to water loss in the samples. The DSC curve of PAM indicates a strong exothermic peak in the range of 200 °C. The peak for PAM-AMPS appeared a little later, whereas PAM-DMAPS showed no exothermic peaks. The result suggested that the heat resistance of PAM-AMPS was higher than that of the other polymers. As can be seen in this curves, the glass transition temperature for the samples are 193–225 °C, corresponds to the second order transition (Table 2).

### Sedimentation test

**Fig. 4:**
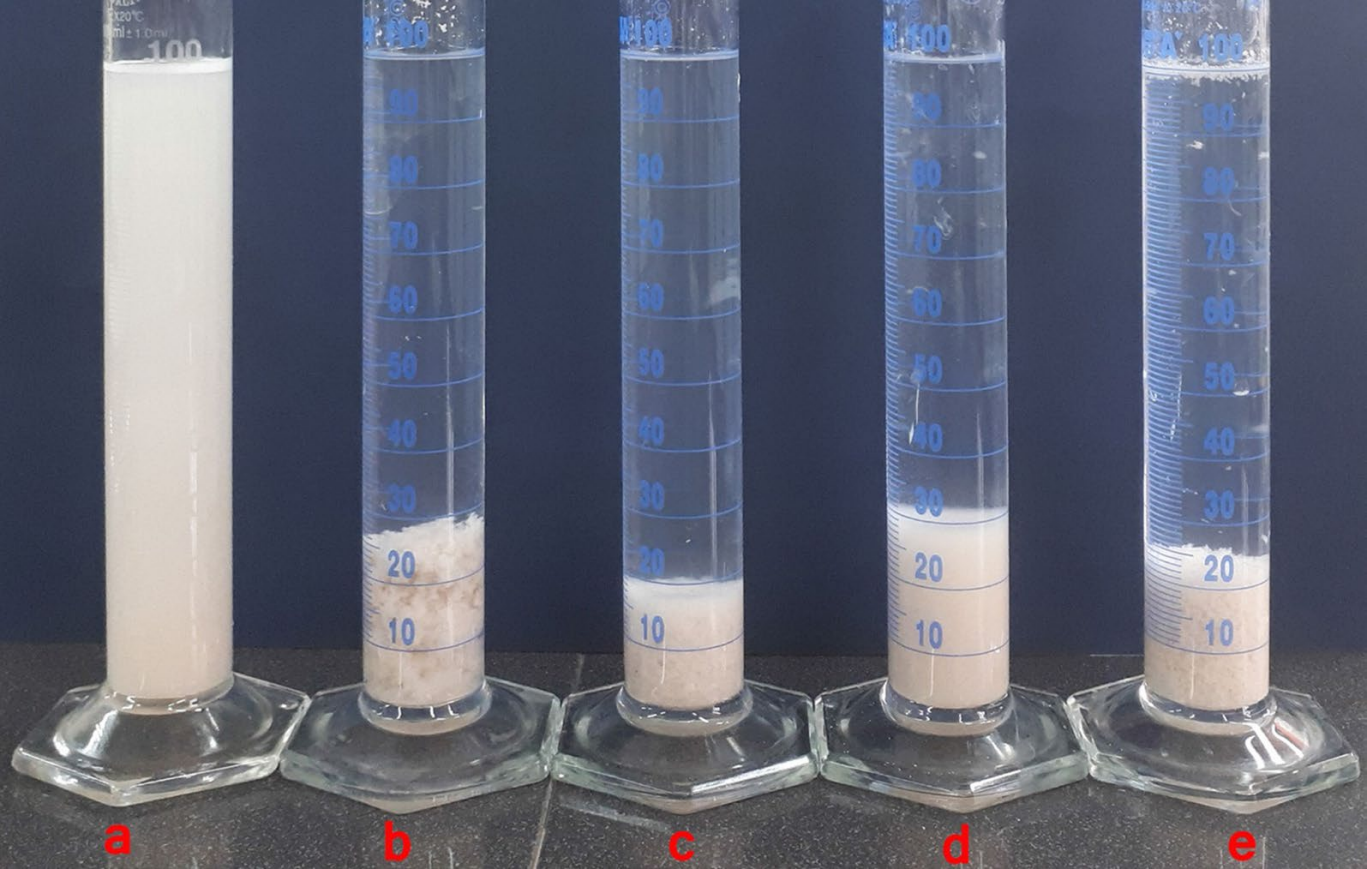
The aggregation and sedimentation behavior of MMT dispersions in different inhibitor solutions: **a** MMT; **b** MMT + PAM; **c** MMT + PAM-DMAPS; **d** MMT + PAM-AMPS; **e** MMT + PAM-DADMAC; (5%, The photo was taken after an hour)

**Scheme 2:**
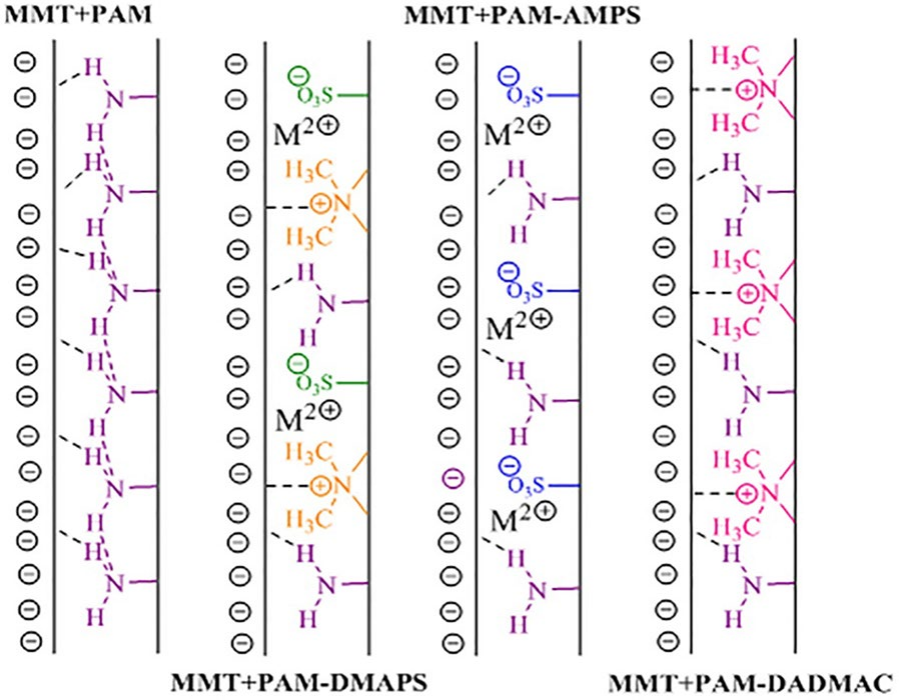
The mechanism of bridging flocculation in different inhibitor solutions

The sedimentation test results show that different electrostatic attractions cause compression and coagulation of MMT (Fig. 4, Scheme 2) [7]. Non-ionic products such as PAM adsorb only via hydrogen bonding. The positive charge created on the non-ionic pendant groups of a polymer allows it to be adsorbed to the negative sites on the surface of the MMT particles. In anionic sample, in addition to hydrogen bonds, salt linkage adsorption is also observed. Salt linkages can be formed between cationic metal ions and the negatively charged pendant groups. Another electrostatic attraction is observed in relation to the cationic coagulant, which has dangling groups that carry the opposite charge on the MMT surface. The amphoteric sample is the best type of coagulant due to having all three types of electrostatic attraction.

### Fourier transform infrared spectrometer of MMT and MMT-copolymer composites

**Fig. 5:**
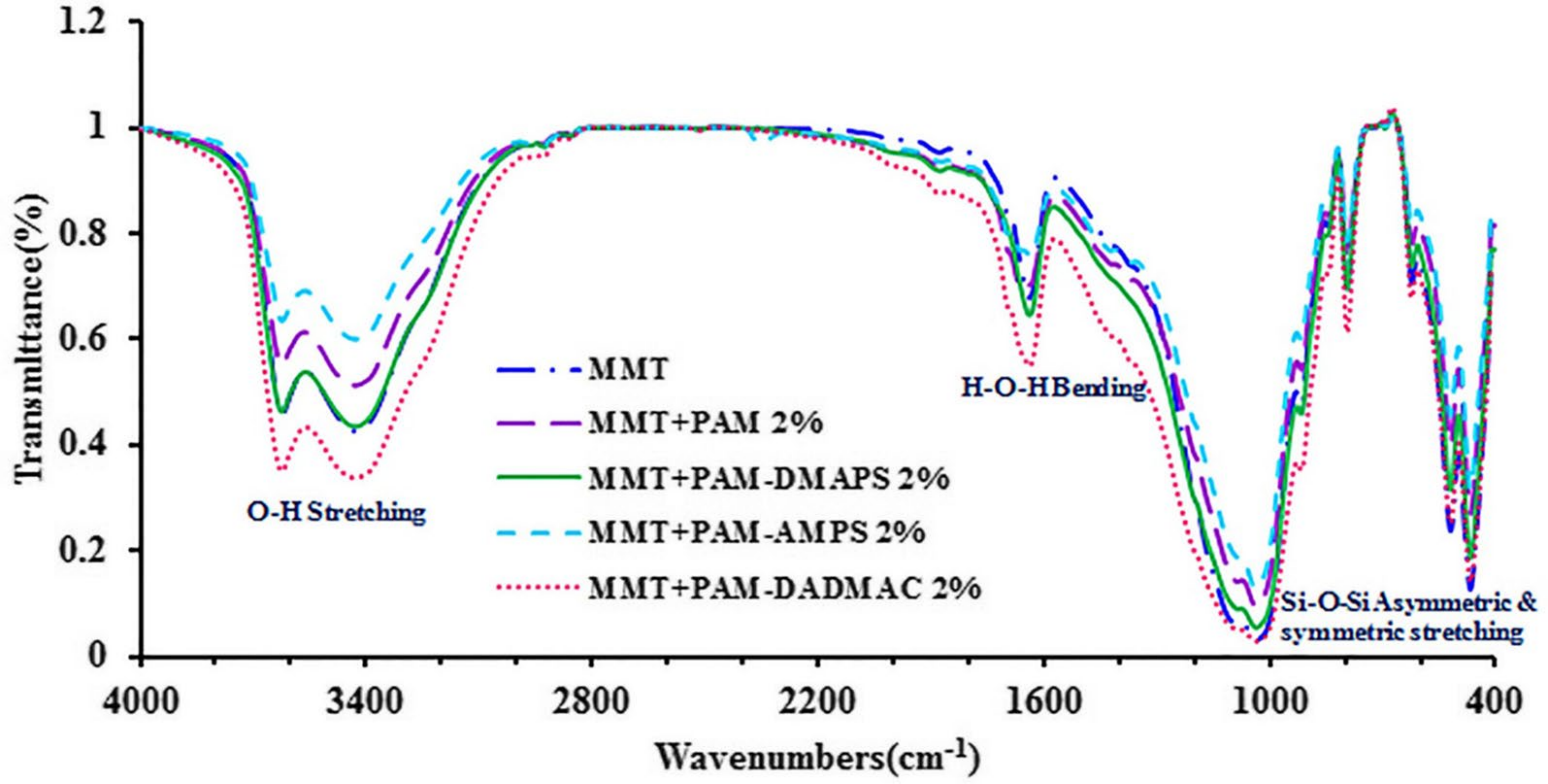
FT-IR spectra of MMT and MMT-copolymer composites

The infrared spectrum of the MMT and MMT-copolymer composites are presented in Fig. 5. The absorption bands at 1032 and 900 cm^−1^ were associated with the symmetric and asymmetric stretch of Si-O-Si bonds of the structure respectively [24, 25]. Also, some other bands appeared at about 3400 and 1620 cm^−1^ which can indicate the presence of hydroxyl groups and the adsorbed water on the surface. FT-IR study does not show significant changes in the structure of polymers after adding MMT in AM matrix based on PAM and copolymers.

### X-ray diffraction analysis

**Fig. 6:**
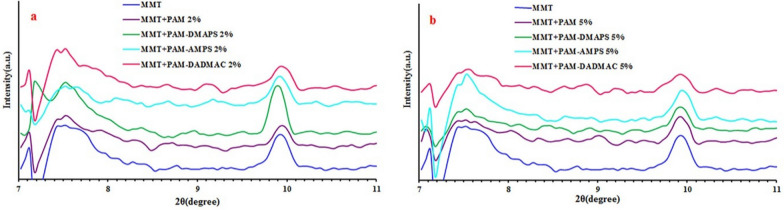
XRD patterns of MMT and MMT-copolymer composites (**a**. 2%, **b**. 5%)

With this technique, the spaces between MMT layers can be determined using Bragg’s formula d = nλ/2sinθ, where λ represents the wavelength of the x-ray and d is the distance between the planes and θ stands for the diffraction angle. XRD patterns of MMT, MMT-PAM and MMT-copolymers (PAM-DMAPS, PAM-AMPS, PAM-DADMAC) composites with different monomer contents are indicated in Fig. 6. A distinct (001) peak at 2θ = 7.546° is displayed for the neat MMT, which suggests a basal plane spacing of 1.171 nm. The XRD pattern shows that the characteristic crystalline peak is shifted to the higher angles for composites in comparison to neat MMT due to the intercalation of PAM, PAM-DMAPS, PAM-AMPS and PAM-DADMAC with the MMT layers (Fig. 6 (a.2%) and (b.5%)) (Table 3) [26, 27]. The reason for the decrease in the distance between the plates in the anionic sample can be related to the capsulation of the MMT, due to its long chain length. In other samples, the distance between the plates remained constant compared to MMT, which indicates the lack of water penetration between the plates.

**Table 3:** X-ray diffraction data of MMT and MMT-copolymer composites

Polymer	2θ (°)	d-spacing (nm)
MMT MMT + PAM 2%	7.546 7.582	1.171 1.170
MMT + PAE-DMAPS 2%	7.543	1.171
MMT + PAM-AMPS 2% MMT + PAM-DADMAC 2% MMT + PAM 5% MMT + PAM-DMAPS 5% MMT + PAM-AMPS 5% MMT + PAM-DADMAC 5%	7.745 7.498 7.491 7.440 7.560 7.480	1.140 1.178 1.179 1.187 1.168 1.181

### Scanning electron microscope analysis

**Fig. 7:**
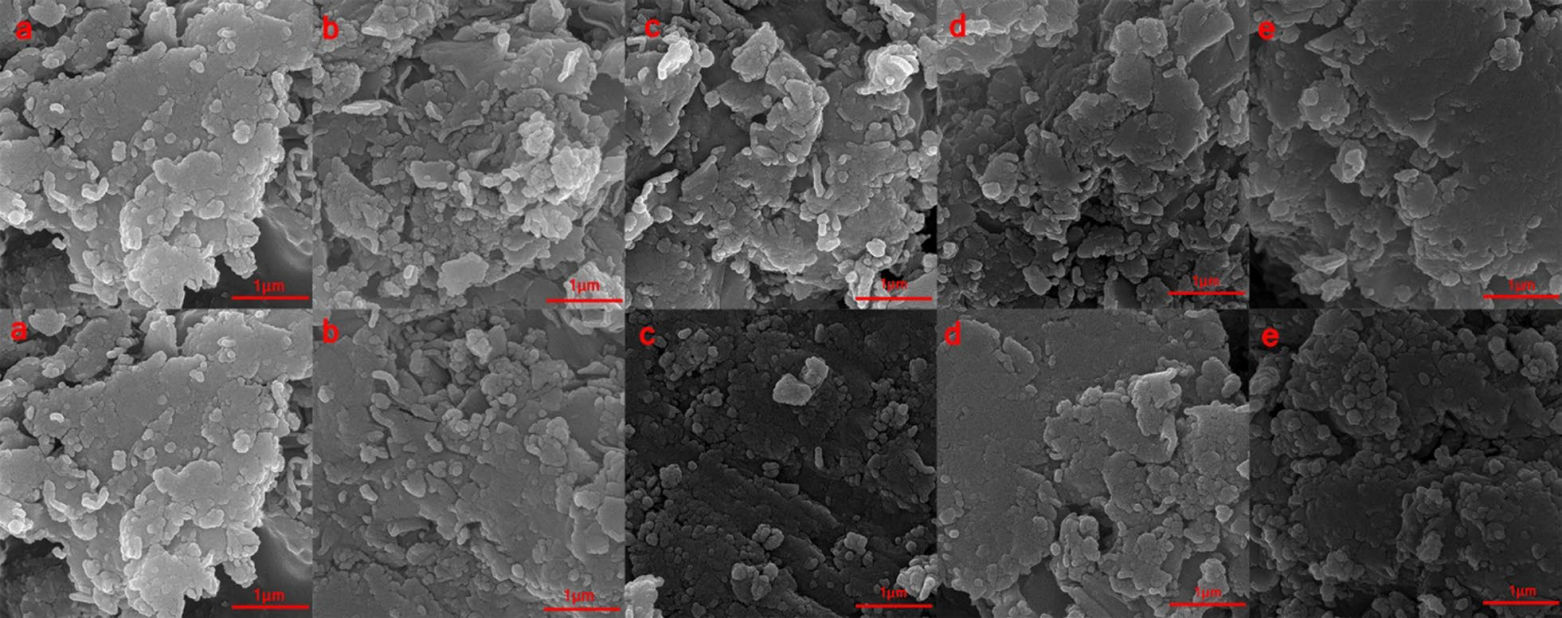
The FE-SEM images of **a** MMT; **b** MMT + PAM; **c** MMT + PAM-DMAPS; **d** MMT + PAM-AMPS; **e** MMT + PAM-DADMAC; (Top images were 2% and bottom images were 5%)

The SEM micrographs of MMT-copolymers composite and free MMT are presented in Fig. 7. The micrographs confirm that the MMT is uniformly dispersed in the polymer matrixes and show several regions of fine structure, especially in the 2% MMT-copolymers samples. MMT + PAM-DMAPS and MMT + PAM-AMPS have more uniform dispersion than others. The results of FT-IR and XRD study explain that the incorporation of MMT with copolymers was properly performed.

### Contact angle measurement

**Fig. 8:**
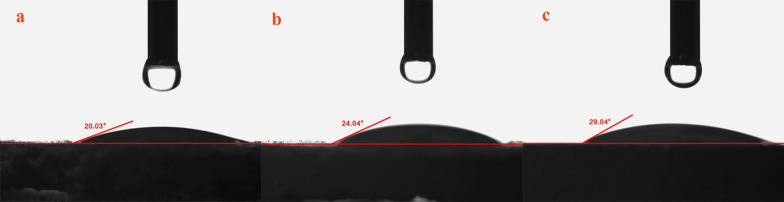
The contact angle measurement of **a** MMT; **b** MMT + PAM; **c** MMT + PAM-DMAPS

Figure 8 shows the results of contact angle for the MMT and some of MMT-copolymers composites. The contact angle of the MMT is 20.03° and for MMT-copolymers is less than 90°, which describes the surface as hydrophilic. The surface contact angle of composites after addition of PAM and copolymers-based AM is increased, which means that copolymers are less hydrophilic compared to MMT. The effect of interlayer d-spacing on the contact angle of montmorillonite used in polymer nanocomposites was studied. As the space d increases, a general increase in contact angles is observed.

## Conclusions

In this study, novel flocculants were synthesized for water-based drilling fluid through polymerization of AM and copolymers-based AM via conventional free radical polymerization technology. FT-IR analysis shows the presence of functional groups in copolymers and proves that AM, DMAPS, AMPS and DADMAC copolymers were successfully synthesized. Furthermore, TGA data reveals that all the homo-and copolymers show two steps degradation and high thermal stability. DSC data show that PAM-AMPS, having a rigid aromatic group, have a higher Tg than other copolymers. Flocculation testing proves that the copolymer-based AM flocculants exhibit comparable results to the PAM flocculants with even better supernatant turbidity in some cases. The homogeneous MMT nanocomposites are formed and characterized by FT-IR, X-ray diffraction and SEM techniques. The SEM and XRD results confirmed that the MMT was homogenously dispersed and exfoliated in the polymer matrixes with an interlayer spacing. Contact angle measurements indicated a reduce of the hydrophilic character for all MMT-copolymers. The PAM and copolymers-based AM are alternatives to other flocculants in water-based drilling fluids.

## Data Availability

All research data are available in the article.
